# miRNA-Mediated Priming of Macrophage M1 Differentiation Differs in Gram-Positive and Gram-Negative Settings

**DOI:** 10.3390/genes13020211

**Published:** 2022-01-24

**Authors:** Georg Riechert, Daniel Maucher, Birte Schmidt, Julia Schumann

**Affiliations:** University Clinic and Outpatient Clinic for Anesthesiology and Operative Intensive Care, University Medicine Halle (Saale), Franzosenweg 1a, 06112 Halle (Saale), Germany; Georg_riechert007@web.de (G.R.); Daniel.Maucher@uk-halle.de (D.M.); birteschmidt09@gmail.com (B.S.)

**Keywords:** miRNA, macrophage, M1 differentiation, Gram-positive, Gram-negative

## Abstract

A proper regulation of macrophage polarization is essential for the organism’s health and pathogen control. Differentiation control is known to occur at the transcriptional as well as the posttranscriptional levels. The mechanisms involved, however, have not yet been fully elucidated. In this study, we co-cultured macrophages with viable Gram-positive and Gram-negative bacteria to mimic macrophage differentiation to the M1-like type in an inflammatory milieu. We found that Gram-positive stimulation resulted in increased expressions of miR-7a-5p, miR-148a-3p, miR-155-5p, and miR-351-5p. Of note, these miRNAs were found to target inhibitory mediators of the Rac1-PI3K-Akt pathway and the MyD88-dependent pathway. In contrast, Gram-negative stimulation-induced downregulation of miR-9-5p, miR-27b-3p, miR-93-5p, and miR-106b-5p is known to target key members of the Rac1-PI3K-Akt pathway and the MyD88-dependent pathway. These results, taken together, point to a fine-tuning of macrophage polarization by TLR-induced changes in macrophage miRNA profiles. Here, the miRNA-mediated priming of M1 differentiation seems to differ in the Gram-positive and Gram-negative settings in terms of the mechanism and miRNAs involved.

## 1. Introduction

Macrophages represent key cellular players of the innate immune system. A major characteristic of these cells is their distinct plasticity. In response to external stimuli and signaling agents, macrophages may differentiate into various phenotypes, which differ substantially in function. The M1-type-like macrophages are pro-inflammatory in nature, producing reactive oxygen intermediates (ROIs) and releasing inflammation-promoting cytokines such as TNF-α, IL-1β, and IFN-γ [1,2,3,4,5]. The M2-type-like macrophages, however, are primarily involved in inflammation resolution and are characterized by a high phagocytotic activity to eliminate cellular debris [1,2,3,4,5]. For the maintenance of the organism’s health as well as for pathogen control, it is essential that macrophage polarization is properly controlled. Dysregulation may be associated with pathological conditions, including both acute and chronic inflammatory processes [1,2,3,5].

Macrophages express various receptors on their cell surface to recognize Gram-positive or -negative pathogens. Toll-like receptors (TLRs) are of utmost importance. Upon interaction with the Gram-positive stimulus lipoteichoic acid (LTA), TLR2 combines with TLR1 or TLR6 to form a heterodimer. Via the adapter proteins ras-related C3 botulinum toxin substrate 1 (Rac1) and myeloid differentiation primary response 88 (MyD88), the signal is transmitted into the cell, inducing activation of the kinases RAC-α serine/threonine-protein kinase (Akt1), IRAK1, and interleucin-1 receptor-associated kinase 1/4 (IRAK4), which ultimately translates into activation of the transcription factors activating protein 1 (AP-1) and nuclear factor kappa-light-chain-enhancer of activated B cells (NFκB) [6,7,8]. Recognition of the Gram-negative stimulus lipopolysaccharide (LPS) is achieved by cluster of differentiation 14 (CD14), which ultimately interacts with TLR4 [6,7,8]. After homodimerization of TLR4, the signal is transduced into the cell via the adapter protein MyD88 [6,7,8]. The kinases IRAK1 and IRAK4 are activated, leading to the initiation of the transcription factors AP-1 and NFκB, analogous to the Gram-positive setting [6,7,8]. As a result, the expression profile of the cell is altered, resulting in differentiation into the M1-like type [4].

The signaling cascades and transcription factors triggered by TLR stimulation, i.e., the transcriptional regulation level, have been thoroughly described. There is, however, substantial uncertainty regarding the control of macrophage polarization at the level of post-transcriptional regulation. The central mediators of post-transcriptional regulation are miRNAs. These non-coding RNA molecules are involved in fine-tuning gene expression by affecting the stability and translational efficiency of protein-coding mRNAs [9,10]. This is based on the interaction of miRNA and mRNA, which implies partial complementarity [9,10]. Accordingly, a single miRNA can influence the expression of hundreds of different mRNAs [9,10].

The aim of the present study was to comparatively determine the miRNA expression profiles of macrophages after Gram-positive or Gram-negative stimulation. Herein, the mode of action of miRNA-based post-transcriptional regulation of macrophage polarization to the M1-like type after TLR stimulation was derived. The study contrasts, for the first time, the effects of Gram-positive and ram-negative bacterial pathogens.

## 2. Materials and Methods

### 2.1. Culturing and Stimulaton of Cells

The murine cell line RAW264.7 (ATCC^®^, Manassas, VA, USA, number TIB-71) was used as a model for monocytes/macrophages. Cells were cultured at 37 °C and 5% CO_2_ in a humidified atmosphere in RPMI 1640 medium containing 4.5 g/L glucose and 5% *v*/*v* FCS. For Gram-positive stimulation of the cells, either lipoteichoic acid (LTA, 0.5 µg/mL, from *Staphylococcus aureus*) or viable virulent *Rhodococcus equi* (ATCC^®^, Manassas, VA, USA, number 33701, bacteria/cell ratio 0.1:1) was added to the cell culture medium for a period of 24 h. Gram-negative stimulation of cells was performed by adding lipopolysaccharide (LPS, 1 µg/mL, from *Escherichia coli* serotype 0111:B4) or viable virulent *Pseudomonas aeruginosa* (ATCC^®^, Manassas, VA, USA, 10145, growth restriction by gentamycin (10 µg/mL), bacteria/cell ratio 1:1). These stimulation conditions have been proven to induce cell differentiation to the M1-like type [11,12,13].

### 2.2. RNA Isolation

A TRIzol-based standard isolation procedure was used to obtain total RNA according to the manufacturer’s instructions (Thermo Fisher Scientific, Dreieich, Germany). The quality of extracted RNA was checked with the NanoDrop spectrophotometer (Thermo Fisher Scientific, Dreieich, Germany) and the Agilent Bioanalyzer (Agilent Technologies, Waldbronn, Germany). Only RNA with an absorbance quotient A260/280 >1.8 was subsequently used for analyses.

### 2.3. Next-Generation Sequencing (NGS)

Expression analysis of miRNAs and the transcriptome (i.e., library preparation and deep sequencing) were performed by Novogene (U.K.) Company Limited (Cambridge, UK) and the Core Unit DNA, Leipzig University (Germany) utilizing an Illumina HiSeq2500 and an Illumina HiScanSQ (San Diego, CA, USA), respectively. Three biological replicates were analyzed in each study group. For miRNA sequencing, the NEB Next^®^ Multiplex Small RNA Library Prep Set for Illumina^®^ (Set 1) kit was used for library preparation. The library was sequenced to a depth of 10 million reads per sample. A single-end 50 bp sequencing strategy was used.

Differential gene expression analysis was performed by the Core Facility Imaging, University Medicine Halle (Germany) and the Core Unit DNA, Leipzig University (Germany). Removal of low-quality read ends as well as remaining parts of sequencing adapters was performed using Cutadapt software (https://cutadapt.readthedocs.org/; accessed on 6 April 2020). Alignment of processed sequencing reads to the murine genome (UCSC mm10) was performed using Bowtie2 v2.3.2 [14] for miRNA analysis and HiSat2 v2.1.0 (http://daehwankimlab.github.io/hisat2/; accessed on 6 April 2020) [15] for transcriptome analysis. For removal of secondary alignments along with filtering and indexing of alignments, samtools [16] was used. Feature-Counts v1.53 (http://subread.sourceforge.net/; accessed on 6 April 2020) [17] was applied for summarizing gene-mapped reads. miRBase v21/22 [18] and Ensembl [19] were utilized as annotation bases. Differential gene expression was analyzed using the R package edgeR v3.26.8 (http://bioconductor.org/packages/release/bioc/html/edgeR.html; accessed on 6 April 2020) [20], utilizing trimmed mean of M-values (TMM) normalization [21] applied to raw read counts. Normalized count data were subsequently transformed into counts per million mapped reads (CPM) for small RNA data and fragments per million imaged reads (FPKM) for polyA RNA data. All generated RNA-seq data were deposited in the Gene Expression Omnibus (GEO) repository.

### 2.4. Droplet Digital PCR (ddPCR)

Complementary DNA (cDNA) was synthesized according to the standard protocol using a miRCURY LNA RT Kit (QIAGEN, Hilden, Germany). The spike-in RNA UniSp6 was added to all samples as the positive control. Copy numbers of specific miRNAs were determined by digital droplet PCR technology (BioRad, Munich, Germany). The manufacturer’s standard protocols, miRCURY LNA miRNA PCR assay primer (QIAGEN, Hilden, Germany), and ddPCR EvaGreen Supermix (Bio-Rad, Munich, Germany) were used. Samples were measured using a QX200 ddPCR Droplet Reader (Bio-Rad, Munich, Germany) to ultimately determine the nucleic acid copy number per nanogram of RNA. For each study group, the ddPCR reaction was performed with five to six biological replicates and two technical replicates. An unpaired t-test was performed to determine significant mean differences, with a *p*-value <0.05 as an indicator of significant differences. The software used for statistical analysis was GraphPad Prism 9 (GaphPad Software, La Jolla, CA, USA). In contrast to classical real-time PCR, which only allows relative quantification, ddPCR precisely determines absolute miRNA copy numbers. A reference RNA is not required for ddPCR, eliminating the risk of normalization-based data bias [22].

### 2.5. In Silico Analyses

A gene set enrichment analysis (GSEA) was performed to determine statistically significant and consistent differences in the transcriptome of Gram-positive-stimulated, Gram-negative-stimulated, or unstimulated macrophages. GSEA was performed by the Core Facility Imaging, University Medicine Halle (Germany). GSEA v3.0 software (UC San Diego and Broad Institute, San Diego, CA, USA) [23] and MSigDB v7.0 gene sets [24] were used and the pre-ranked test, 1000 permutations, and classical scoring scheme were applied. The log2 fold changes in expression of all protein-coding genes determined by the differential expression analyses were input. A false discovery rate (FDR) <0.05 was set as a cutoff criterion.

Transcription factors known to be activated as a result of TLR stimulation were determined using the KEGG online database (https://www.genome.jp/kegg/; accessed on 27 May 2021) [6].

Identification of transcription factor binding sites of the differentially expressed miRNAs was performed using the GeneCards v5.7 online database (https://www.genecards.org/; accessed on 27 May 2021) [25].

Target genes of differentially expressed miRNAs were identified utilizing the online database miRWalk (http://mirwalk.umm.uni-heidelberg.de/; accessed on 29 April 2021) [26].

Gene Ontology (GO) enrichment analysis was performed on the identified target genes of each miRNA using the Cytoscape plugin ClueGO v2.5.7 (https://apps.cytoscape.org/apps/cluego; accessed on 29 April 2021) [27]. A false *p*-value <0.05, as determined after Bonferroni correction, was set as a cutoff criterion.

## 3. Results

### 3.1. Gram-Positive Stimulation of Macrophages Induces Upregulation of miR-7a-5p, miR-148a-3p, miR-155-5p, and miR-351-5p, Whereas Gram-Negative Stimulation of Macrophages Induces Downregulation of miR-9-5p, miR-27b-3p, miR-93-5p, and miR-106b-5p

**Figure 1:**
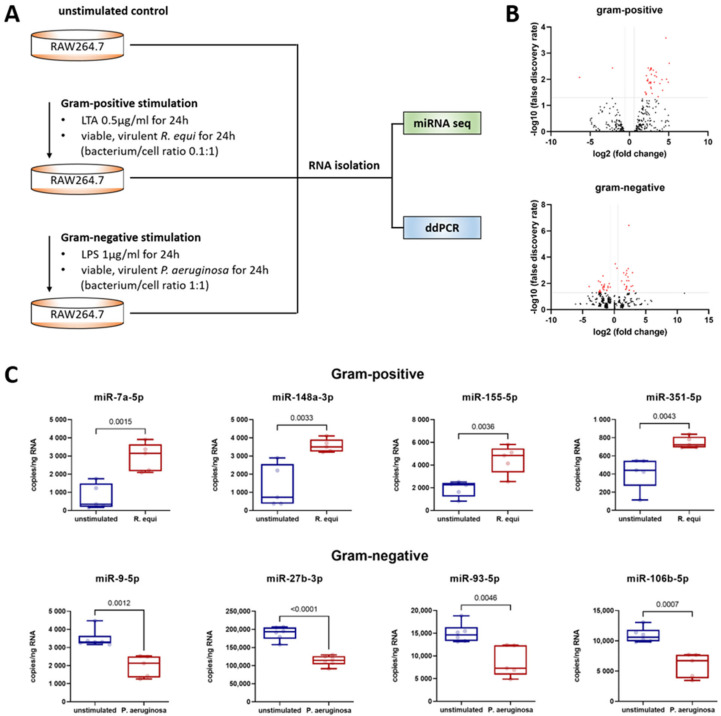
Murine macrophages (cell line RAW264.7, ATCC^®^ number TIB-71) were evaluated following Gram-positive or -negative stimulation for 24 h and compared to unstimulated controls. (**A**) Workflow showing the cell culture conditions for miRNA sequencing and ddPCR. (**B**) Volcano plots showing the distribution of the fold changes and false discovery rates of all annotated miRNAs in a comparison between unstimulated and stimulated macrophages. For stimulation, either LTA (Gram-positive setting) or LPS (Gram-negative setting) was used. Red points above the horizontal line mark significant miRNAs (FDR > 0.05). The vertical lines mark the effect size threshold used. (**C**) Absolute miRNA quantification was performed by ddPCR analysis of isolated total RNA utilizing a QX200 Droplet Digital PCR System (Bio-Rad) and specific LNA PCR primers (Qiagen). The copy number of the target RNA in the total mixture was calculated by the system based on the number of positive droplets measured and assuming a Poisson distribution. In each ddPCR assay, a specific amount of RNA was used so that the copy number per ng of RNA can be calculated. The jittered box plots depict the median, the lower and upper quantile, the two extreme values, and the individual sample’s data points. Each group included five to six biological replicates and two technical replicates. Significant differences were identified utilizing an unpaired *t*-test. Statistical analysis was performed using GraphPad Prism 9 (GaphPad Software, La Jolla, CA, USA). A *p*-value < 0.05 was assumed to indicate significant differences. The *p*-values are specified in each diagram.

A next-generation sequencing (NGS)-based screening approach was used to assess changes in macrophage miRNA expression mediated by a Gram-positive (i.e., LTA) or Gram-negative (i.e., LPS) stimulus (Figure 1). The complete data set can be found in the GEO repository (accession numbers GSE162995 and GSE132361). All miRNAs, which showed (i) an abundance >100 reads, (ii) a stimulation-induced altered expression rate >1.5/<0.67-fold, and (iii) an effect size (Cohen’s D) >0.8/<−0.8 were subjected to a PCR-based validation. Here, to mimic the physiological response of macrophages to pathogens, stimulation of cells was performed by co-culture with viable, Gram-positive (i.e., *R. equi*) or Gram-negative (i.e., *P. aeruginosa*) bacteria. For validation, absolute quantification by digital droplet PCR (ddPCR) was performed.

As shown in Figure 1, Gram-positive stimulation of macrophages resulted in the upregulation of miR-7a-5p, miR-148a-3p, miR-155-5p, and miR-351-5p. Compared to the unstimulated control, macrophages maintained in co-culture with the Gram-positive bacterium *R. equi* for 24 h displayed an increase in copy number of 4.0-fold for miR-7a-5p, 2.7-fold for miR-148a-3p, 2.4-fold for miR-155-5p, and 1.8-fold for miR-351-5p. In contrast, Gram-negative stimulation of macrophages resulted in the downregulation of miR-9-5p, miR-27b-3p, miR-93-5p, and miR-106b-5p (Figure 1). Compared to the unstimulated control following co-culture with the Gram-negative bacterium *P. aeruginosa* for 24 h, copy numbers were reduced to 0.6-fold for miR-9-5p, miR-27b-3p, and miR-93-5p, and to 0.5-fold for miR-106b-5p.

To obtain a full picture, the miRNAs with altered expression after Gram-positive stimulation were also examined by ddPCR in samples gained from macrophages exposed to a Gram-negative setting (and vice versa). Here, no effect on copy number was detected in any case. This suggests that post-transcriptional regulation after TLR1/2/6 or TLR4 stimulation is driven by distinct miRNAs.

### 3.2. In Both Gram-Positive and Gram-Negative Settings, the miRNAs with Alterd Expression Are Subject to the Influence of Transcription Factors Triggered by TLR Signaling

Control of miRNA expression is achieved at the transcriptional level by the interaction of transcription factors with specific binding sites on the miRNA-coding region of DNA. To identify the transcription factors that may mediate the altered miRNA expression after TLR stimulation, a screen was performed in the GeneCards v5.7 database. The transcription factor binding sites listed in GeneCards v5.7 were aligned to the transcription factors designated as TLR-associated in the KEGG database. All transcription factors fulfilling both criteria are listed in Table 1. Numerous hits were obtained for AP-1 and AP-1-interacting transcription factors. Given these in silico data, it seems reasonable to assume that the miRNA expression changes downstream TLR stimulation are mediated by AP-1.

### 3.3. Altered miRNA Profiles Promote Macrophage Polarization by Distinct Mechanisms of Action in Gram-Positive and Gram-Negative Settings

**Figure 2:**
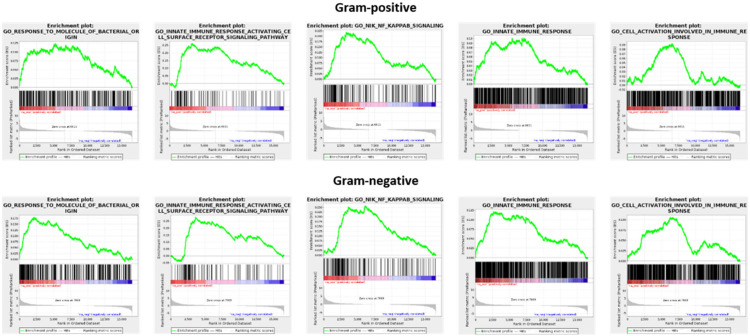
Gene set enrichment analysis (GSEA) applied to expression changes of protein-coding genes found in the comparison of unstimulated and stimulated macrophages. For stimulation, either LTA (Gram-positive setting) or LPS (Gram-negative setting) was used.

GSEA analysis was used to compare the transcriptomes of unstimulated and stimulated macrophages (GEO repository accession numbers GSE162994 and GSE142088). Consistently, both Gram-positive and -negative stimulation were associated with a significant enrichment (FDR < 0.05) of distinct gene sets, namely GO:0002237 (response to molecule of bacterial origin), GO:0002220 (innate immune response activating cell surface receptor signaling pathway), GO:0038061 (NIK/NF-kappaB signaling), GO:0045087 (innate immune response), and GO:0002263 (cell activation involved in immune response) (Figure 2). This underscores that following recognition of both Gram-positive and -negative bacteria, there are common alterations at the mRNA level that ultimately facilitate differentiation to the M1-like type.

To determine whether the stimulation-induced alterations in the miRNA profile may contribute to macrophages polarization, the corresponding target genes were identified in silico utilizing the miRWalk database. The entire list of target genes (both experimentally validated and predicted due to sequence homologies) of miRNAs expressed altered after Gram-positive or -negative stimulation were subject to a GO enrichment analysis by ClueGO. Remarkably, despite the fundamental differences in the miRNA profiles after Gram-positive or -negative stimulation, identical GO terms were found to be significantly enriched. They included GO:0048583 (regulation of response to stimulus), GO:0023051 (regulation of signaling), GO:0009966 (regulation of signal transduction), GO:0010468 (regulation of gene expression), and GO:0045595 (regulation of cell differentiation). The results of GO enrichment analysis provide a clear indication that both the upregulation of miRNAs miR-7a-5p, miR-148a-3p, miR-155-5p, and miR-351-5p observed after Gram-positive stimulation and the downregulation of miRNAs miR-9-5p, miR-27b-3p, miR-93-5p, and miR-106b-5p observed after Gram-negative stimulation are ultimately related to macrophage polarization.

In a further analysis step, the gene lists of the individual miRNAs determined by miRWalk were analyzed for relevant target genes. As shown in Table 2, numerous mediators of TLR signaling were identified; yet, distinct differences between the Gram-positive and -negative settings became apparent. Target genes of miRNAs upregulated after Gram-positive stimulation include inhibitors of NFκB activation (e.g., CHUK and NFKBIB) and mediators of M2 differentiations (e.g., AKT1, CREB1, CEBPB, SOCS1, and FOSL2). Post-transcriptional expression inhibition of these genes resulting from upregulation of miR-7a-5p, miR-148a-3p, miR-155-5p, and miR-351-5p fits well within the established M1 polarization response to TLR2 stimulation. In contrast, miRNAs downregulated after Gram-negative stimulation were found to primarily target central members of the TLR4 signaling cascade. This indicates a reinforcing feedback loop. Responding to TLR4 stimulation, there was downregulation of miR-9-5p, miR-27b-3p, miR-93-5p, and miR-106b-5p, which in turn may be associated with attenuation of post-transcriptional inhibition of TLR4-induced signaling cascades.

## 4. Discussion

Certain cell wall components of Gram-positive and Gram-negative bacteria, namely LTA and LPS, are well-known to trigger M1 polarization of macrophages by stimulating the membrane receptors of the Toll-like family [6,7,8]. Major signaling cascades involved are the Rac1–PI3K–Akt pathway and the MyD88-dependent pathway, which regulate the activation of transcription factors such as AP-1 and NFκB [6,7,8]. Transcription factors not only influence the expression of protein-coding genes but also the expression of regulatory RNAs, one of which is the miRNAs [9,10]. However, while transcriptional regulation of macrophage response to bacterial stimuli has been well-described, there is only limited information focusing on post-transcriptional control events. A comparative view of macrophage miRNA profiles induced by Gram-positive and -negative stimulation has not yet been described in the scientific literature.

In the Gram-positive setting simulated by LTA or co-culture with *R. equi*, marked upregulation of miR-7a-5p, miR-148a-3p, miR-155-5p, and miR-351-5p was found. Increased expression of miR-155-5p has already been described in previous studies as a consequence of macrophage stimulation with the Gram-positive pathogens *S. pneumoniae*, *L. monocytogenes*, and *M. bovis* [28,29,30]. All these findings suggest that a generalizable effect of Gram-positive bacteria on macrophages is upregulating miR-155-5p. Moreover, the present study is the first to demonstrate that miRNAs miR-7a-5p, miR-148a-3p, and miR-351-5p can be regulated by TLR2. The findings are supported by the mapping of TLR2-activated transcription factors to the transcription binding sites of the miRNAs. Striking is an accumulation of subunits of the transcription factor AP-1 (i.e., FOS, FOSL2, JUN, JUNB, JUND) [6] as well as of transcription factors that are interrelated with AP-1. These include the transcription factors ATF4, NFATC1, and NFATC3, which co-operate with AP-1 [31,32,33]; the transcription factors BCL6 and BCL6B, which mediate the repression of AP-1 activity [34]; and the transcription factors MAX and MYC, whose expression is affected by AP-1 [35,36]. Furthermore, the in silico analyses provide strong indications that miR-7a-5p, miR-148a-3p, miR-155-5p, and miR-351-5p are involved in the regulation of macrophage polarization. First, GO enrichment analysis points in this direction. Second, database entries show that all four miRNAs target the expression of key inhibitory mediators of the Rac1–PI3K–Akt pathway and the MyD88-dependent pathway. Interaction of miRNAs with (partially) complementary mRNAs is usually accompanied by decreased mRNA stability and/or translation efficiency. Consequently, upregulation of miR-7a-5p, miR-148a-3p, miR-155-5p, and miR-351-5p after Gram-positive stimulation is expected to be linked to post-transcriptional silencing of the specific target genes. Strikingly, target genes of miRNAs upregulated after Gram-positive stimulation include *CHUK* and *NFKBIB*, which are known to block NFκB activation [6]. The net result would be an attenuation of NFκB inhibition. Attenuation of the expression of the target gene *FOS* was shown to be linked to an increase in TLR expression [37]. Further identified target genes include *AKT1*, *CREB1*, *CEBPB*, *SOCS1*, and *FOSL2*, which have been reported to mediate M2 differentiation [38,39,40,41,42,43,44] and *NFAT*, which is known for having a generically inhibitory effect on macrophage differentiation [45]. Hence, post-transcriptional repression of these target genes by miRNAs upregulated after Gram-positive stimulation reasonably favors differentiation of macrophages to the M1-like type. In line with these data, previous observations also showed that knockout of miR-155-5p in macrophages was associated with the impaired clearance of *S. pneumoniae* and *M. bovis* [28,30].

In the Gram-negative setting simulated by LPS or co-culture with *P. aeruginosa*, marked downregulation of miR-9-5p, miR-27b-3p, miR-93-5p, and miR-106b-5p was found. The consequences of Gram-negative stimulation on the miRNA profile of macrophages have been more extensively studied than the Gram-positive situation. Accordingly, there is already evidence in the scientific literature of expression changes of miR-9-5p, miR-27b-3p, and miR-93-5p after treatment of macrophages with LPS [46,47,48,49]. However, the data presented here are the first to show downregulation of these miRNAs following physiological contact between macrophages and viable Gram-negative bacteria. The data also reveal a clear difference in the miRNA profiles of macrophages after Gram-positive and -negative stimulation. This is all the more remarkable given that mapping of transcription factors activated by TLR4 to the miRNA’s transcription factor binding sites revealed definite matches for AP-1 and AP-1-associated transcription factors, analogous to the Gram-positive setting. It is reasonable to assume that miRNAs expressed in an altered manner after Gram-negative stimulation, analogous to the Gram-positive setting, are involved in the regulation of macrophage differentiation to the M1-like type. This is indicated by the results of GO enrichment analysis. Furthermore, it has been well-documented both in the literature and database entries that miR-9-5p, miR-27b-3p, miR-93-5p, and miR-106b-5p target key mediators of the Rac1–PI3K–Akt and the MyD88-dependent pathways [46,47,48,49]. Downregulation of miR-9-5p, miR-27b-3p, miR-93-5p, and miR-106b-5p after Gram-negative stimulation should translate into increased expression of the specific target genes. This suggests that in the presence of Gram-negative pathogens macrophage polarization to the M1-like type is favored via the abrogation of post-transcriptional repression of those target genes and a resultant potentiation of the TLR4 signaling pathway.

Taken together, this study highlights the possibility that TLR-induced changes in macrophage miRNA profiles are involved in fine-tuning macrophage polarization. For this task, the mechanism of miRNA-mediated priming of M1 differentiation seems to differ in the Gram-positive and G-negative settings and to be based on separate miRNAs.

## Figures and Tables

**Table 1:** Transcription factors that (i) are subject to influence by TLR signaling pathways and (ii) are capable of interacting with the promoter region of miRNAs altered after TLR stimulation as identified using the databases GeneCards v5.7 and KEGG. “+” indicates that the transcription factor binds the transcription factor binding site of the miRNA; “−” indicates non-binding.

Transcription Factor	miR- 7a-5p	miR- 148a-3p	miR- 155-5p	miR- 351-5p	miR- 9-5p	miR- 27b-3p	miR- 93-5p	miR- 106b-5p
	Gram-Positive Setting				Gram-Negative Setting			
ATF4	−	+	−	−	+	+	−	−
BCL6	+	+	−	−	+	+	−	−
BCL6B	+	−	−	−	+	+	−	−
ELK1	−	−	−	−	+	−	−	−
FOS	+	+	−	−	+	+	+	+
FOSL2	+	+	−	−	+	+	−	−
JUN	−	+	−	−	+	+	−	−
JUNB	+	+	+	−	+	−	−	−
JUND	+	+	+	−	+	+	−	−
MAX	+	+	+	−	+	+	+	+
MYC	+	+	−	−	+	+	−	−
NFATC1	+	+	+	−	+	−	−	−
NFATC3	+	−	−	−	+	−	−	−

ATF4 = activating transcription factor 4, BCL = B-cell lymphoma, ELK1 = ETS like-1 protein, FOS = fos proto-oncogene, FOSL = FOS like, JUN = jun proto-oncogene, MAX = MYC associated factor X, MYC = myc proto-oncogene, NFATC = nuclear factor of activated T cells.

**Table 2:** Target genes of miRNAs that were altered in expression after TLR stimulation were identified using the miRWalk database.

miRNA	Target Genes Associated with TLR Signaling
Gram-positive setting	
miR-7a-5p	*CREB1, FOS, NFATC1*
miR-148a-3p	*CHUK*
miR-155-5p	*AKT1, CEBPB, FOS, FOSL2, SOCS1*
miR-351-5p	*NFKBIB*
Gram-negative setting	
miR-9-5p	*NFKB1, RELA, TAB2, TAB3*
miR-27b-3p	*MAP3K14, TAB2, TAB3, TRAF6*
miR-93-5p	*IRAK4, MAP3K7, MAP3K14, TAB1, TAB3, TLR4*
miR-106b-6p	*MAP3K14, TAB2, TRAF6*

AKT1 = AKT serine/threonine kinase 1, CEBPB = CCAAT enhancer binding protein β, CHUK = component of inhibitor of nuclear factor kappa B kinase complex, CREB1 = cAMP responsive element binding protein 1, FOS = fos proto-oncogene, FOSL2 = FOS like 2, IRAK4 = interleucin-1 receptor-associated kinase 4, MAP3K7 = mitogen-activated protein kinase kinase kinase 7 (TAK1), MAP3K14 = mitogen-activated protein kinase kinase kinase 14 (=NIK), NFATC1 = nuclear factor of activated T cells 1, NFKB1 = nuclear factor kappa b subunit 1, NFKBIB = NFKB inhibitor β, RELA = nuclear factor kappa b subunit p65, SOCS1 = suppressor of cytokine signaling 1, TAB = TGF-β activated kinase 1 (TAK1/MAP3K7) binding protein, TLR4 = Toll-like receptor 4, TRAF6 = TNF receptor associated factor 6.

## Data Availability

Deep sequencing datasets generated and analyzed in the present study are available in the Gene Expression Omnibus (GEO) repository, accession numbers GSE162995 (miRNA screening after gram-positive stimulation), GSE162994 (transcriptome screening after gram-positive stimulation), GSE132361 (miRNA screening after gram-negative stimulation), GSE142088 (transcriptome screening after gram-negative stimulation).
